# Trends in frailty in brain tumor care during the COVID-19 pandemic in a nationwide hospital network in Germany

**DOI:** 10.1007/s41999-023-00880-0

**Published:** 2023-11-13

**Authors:** Bujung Hong, Ali Allam, Oliver Heese, Rüdiger Gerlach, Hussain Gheewala, Steffen K. Rosahl, Michael Stoffel, Yu-Mi Ryang, Ralf Burger, Barbara Carl, Rudolf A. Kristof, Thomas Westermaier, Jorge Terzis, Farid Youssef, Ralf Kuhlen, Sven Hohenstein, Andreas Bollmann, Julius Dengler

**Affiliations:** 1 Department of Neurosurgery, HELIOS Hospital Bad Saarow, Bad Saarow, Germany; 2 Department of Neurosurgery, Hannover Medical School, Hannover, Germany; 3 Department of Anesthesiology and Intensive Care Medicine, HELIOS Hospital Bad Saarow, Bad Saarow, Germany; 4 Department of Neurosurgery, HELIOS Hospital Schwerin, Schwerin, Germany; 5 Department of Neurosurgery, HELIOS Hospital Erfurt, Erfurt, Germany; 6 Present Address: Faculty of Health Sciences Brandenburg, Brandenburg Medical School Theodor Fontane, Campus Bad Saarow, Pieskower Strasse 33, 15526 Bad Saarow, Germany; 7 Department of Neurosurgery, HELIOS Hospital Krefeld, Krefeld, Germany; 8 Department of Neurosurgery and Center for Spine Therapy, HELIOS Hospital Berlin Buch, Berlin, Germany; 9 Department of Neurosurgery, Klinikum Rechts der Isar, Technical University Munich, Munich, Germany; 10 Department of Neurosurgery, HELIOS Hospital Uelzen, Uelzen, Germany; 11 Department of Neurosurgery, University of Marburg, Marburg, Germany; 12 Marburg Center for Mind, Brain and Behavior (MCMBB), Marburg, Germany; 13 Department of Neurosurgery, HELIOS Dr. Horst Schmidt Kliniken, Wiesbaden, Germany; 14 Department of Neurosurgery, HELIOS Hospital Meiningen, Meiningen, Germany; 15 Department of Neurosurgery, HELIOS Hospital Dachau, Dachau, Germany; 16 Department of Neurosurgery, HELIOS University Hospital Wuppertal, Wuppertal, Germany; 17 Department of Neurosurgery, HELIOS Hospital Plauen, Plauen, Germany; 18 HELIOS Health GmbH, Berlin, Germany; 19 Real World Evidence and Health Technology Assessment, Helios Health Institute, Berlin, Germany; 20 Department of Electrophysiology, Heart Center Leipzig, Leipzig, Germany

**Keywords:** Brain tumor, COVID-19, SARS-CoV-2, Frailty, Hospital Frailty Risk Score, Mortality rates

## Abstract

**Purpose:**

Among brain tumor patients, frailty is associated with poor outcomes. The COVID-19 pandemic has led to increased frailty in the general population. To date, evidence on changes in frailty among brain tumor patients during the pandemic is lacking. We aimed to compare frailty among brain tumor patients in Germany during the COVID-19 pandemic to the pre-pandemic era and to assess potential effects on brain tumor care.

**Methods:**

In this retrospective observational study, we compared frailty among brain tumor patients hospitalized during the COVID-19 pandemic in years 2020 through 2022 to pre-pandemic years 2016 through 2019 based on administrative data from a nationwide network of 78 hospitals in Germany. Using the Hospital Frailty Risk Score (HFRS), frailty was categorized as low, intermediate, or high. We examined changes in frailty, patient demographics, the burden of comorbidity, rates of surgery, and mortality rates for different frailty groups during the pandemic and compared them to pre-pandemic levels.

**Results:**

Of the 20,005 included hospitalizations for brain tumors, 7979 were during the pandemic (mean age 60.0 years (± 18.4); females: 49.8%), and 12,026 in the pre-pandemic period (mean age: 59.0 years [± 18.4]; females: 49.2%). Average daily admissions decreased from 8.2 (± 5.1) during pre-pandemic years to 7.3 (± 4.5) during the pandemic (*p* < 0.01). The overall median HFRS decreased from 3.1 (IQR: 0.9–7.3) during the pre-pandemic years to 2.6 (IQR: 0.3–6.8) during the pandemic (*p* < 0.01). At the same time, the Elixhauser Comorbidity Index (ECI) decreased from 17.0 (± 12.4) to 16.1 (± 12.0; *p* < 0.01), but to a larger degree among high compared to low frailty cases (by 1.8 vs. 0.3 points; *p* = 0.04). In the entire cohort, the mean length of stay was significantly shorter in the pandemic period (9.5 days [± 10.7]) compared with pre-pandemic levels (10.2 days [± 11.8]; *p* < 0.01) with similar differences in the three frailty groups. Rates of brain tumor resection increased from 29.9% in pre-pandemic years to 36.6% during the pandemic (*p* < 0.001) without differences between frailty levels. Rates of in-hospital mortality did not change during the pandemic (6.1% vs. 6.7%, *p* = 0.07), and there was no interaction with frailty.

**Conclusion:**

Even though our findings are limited in that the HFRS is validated only for patients ≥ 75 years of age, our study among patients of all ages hospitalized for brain tumors in Germany suggests a marked decrease in levels of frailty and in the burden of comorbidities during the COVID-19 pandemic.

## Key summary points

**Aim:**

To compare frailty among brain tumor patients in Germany during the COVID-19 pandemic to the pre-pandemic era and to assess potential effects on brain tumor care.

**Findings:**

Using the Hospital Frailty Risk Index, we found that the overall frailty decreased significantly during the COVID-19 pandemic, compared to pre-pandemic levels. The simultaneous decrease in the Elixhauser Comorbidity Index was significantly more pronounced among high compared to low frailty cases.

**Message:**

Among patients hospitalized for brain tumors in Germany, levels of frailty and the burden of comorbidities decreased during the COVID-19 pandemic.

## Introduction

The treatment of brain tumors can be associated with poor outcomes due to neurological deficits, repeat surgical treatment, and adverse reactions to chemoradiotherapy [1, 2]. As in numerous other diseases, another important predictor of outcomes is frailty, which is an age-dependent syndrome defined by high vulnerability to low-power stressors and multimorbidity [3–6].

During the COVID-19 pandemic, lockdown measures and other restrictions led to reduced activity in the general population and a measurable increase in frailty among community-dwelling older persons [7, 8]. To date, there is no large-scale evidence on changes in frailty among brain tumor patients during the COVID-19 pandemic and whether longitudinal trends in frailty may have impacted brain tumor care.

Frailty is reliably quantified using the Hospital Frailty Risk Score (HFRS), a recently introduced scale based on a predefined set of administrative data [9]. Among patients with brain tumors, the HFRS is associated with postoperative complications, length of hospital stay, non-routine discharge disposition, 30-day readmission, and mortality [1, 4, 5, 10–13].

In this study, we aimed to compare frailty among brain tumor patients hospitalized during the COVID-19 pandemic, in years 2020–2022, to corresponding pre-pandemic years 2016–2019 using data from a nationwide network of 78 hospitals in Germany. For different frailty groups, changes in patient demographics, types of management, and in-hospital mortality rates were examined.

## Methods

Administrative data from 78 Helios Hospitals in Germany involved in the diagnosis and treatment of brain tumors were analyzed. Managing 7% of all in-hospital cases and 10% of hospitalized COVID-19 cases nationwide, the Helios hospital network is the largest private healthcare provider in Germany, with centers in rural and urban regions in 13 of the 16 states of Germany [14].

Patients with brain tumors were grouped based on the time of hospital admission, as follows: pre-pandemic years: January 1, 2016–December 31, 2019, and pandemic years: January 1, 2020–December 31, 2022. We identified patients with brain tumors according to the categorization introduced by the AANS/CNS section as a part of the Quality Outcomes Database Tumor Registry [15], using the following International Classification of Diseases, 10th Revision (ICD-10) groups: intracranial metastases (C79.3); primary meningeal tumors (C70.0, D32.0); primary high-grade/malignant brain tumors (C71.0–C71.9); primary low-grade/benign brain tumors (D33.0–D33.2); and pituitary tumors (C75.1; D35.2; D44.3). The types of management were examined using the following operating procedures (OPS) codes categories: craniotomy (5-010 and 5-012); brain tumor resection (5-015, 5-016, and 5-017); transfer to intensive care unit (8-980, 8-98d, 8-98f); and initiation of mechanical ventilation (8-70x, 8-71x). The HFRS was calculated retrospectively for every included case based on ICD-10-Codes, as previously described [9]. Included cases were divided into previously established frailty groups, as follows: low (HFRS below 5 points), intermediate (HFRS 5–15 points), and high (HFRS above 15 points).

The study was approved by the Ethics Committee of the University of Leipzig on February 07, 2022 (490/20-ek). Since this study is observational and presents no identity of enclosed patients, individual informed consent was waived.

### Statistical analysis

Administrative data were extracted using QlikView (QlikTech, Radnor, Pennsylvania, USA). Inferential statistics were based on generalized linear mixed models (GLMM) specifying hospitals as random factor [16]. The effects were estimated with the lme4 package (version 1.1–21) [17] in the R environment for statistical computing (version 4.0.2, 64-bit build) [18]. In all mixed models, we specified varying intercepts for the random factor. For all tests, we apply a two-tailed 5% error criterion for significance. Trends in weekly admission were assessed based on incidence rate and linear regression models.

For the description of the patient characteristics of the cohorts, we employed *χ*2-tests for binary variables and analysis of variance for numeric variables. We report proportions, means, standard deviations, and *p* values.

For the comparison of proportions of selected treatments and outcomes in the different cohorts, we used logistic GLMMs with logit link function. We report proportions and odds ratios together with confidence intervals and *p* values.

Daily case numbers and frailty scores were analyzed with negative binomial models. For the analysis of frailty, we multiplied the scores with ten in order to achieve integer values. We report ratios which are calculated by exponentiation of the regression coefficients together with 95% confidence intervals (CI) for the comparisons of the two periods and *p* values.

The Elixhauser comorbidity index (ECI) and its items were calculated as previously reported [19]. For the weighted ECI, the AHRQ algorithm was applied.

The analysis of the outcome variable length of stay was performed via LMM based on a log-transformed dependent variable. We report means, standard deviations, medians, interquartile ranges (IQR), and *p* values. For all analyses, a p value of  ≤  0.05 was considered significant.

For assessment of the impact of frailty groups on the differences between pre-pandemic and pandemic periods, interaction analyses were used. In other words, by means of interaction analyses, we examined whether changes in certain variables between pre-pandemic and pandemic levels differed across frailty groups, using the high frailty group as reference category. Frailty groups entered the analyses as treatment contrasts (dummy coding; low vs. high, intermediate vs. high), while the period was specified as 0.5 (pandemic) vs. − 0.5 (pre-pandemic).

## Results

**Fig. 1:**
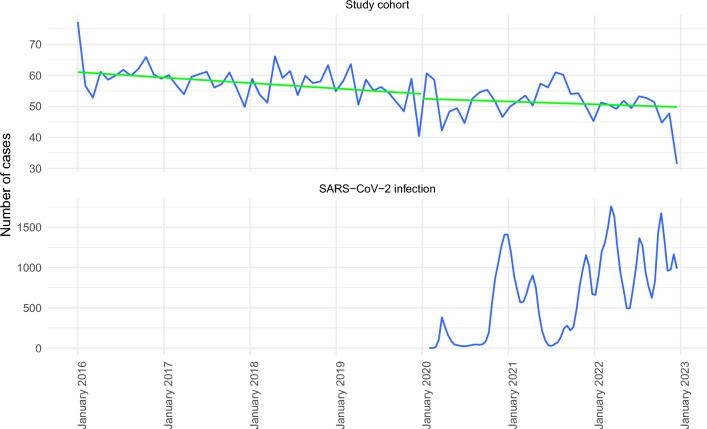
Weekly admissions of patients with brain tumors during the pandemic and pre-pandemic periods in correlation to the SARS-CoV-2 infections (smoothed curves). The green lines depict trends in weekly admission based on linear regression models

A total of 20,005 hospitalizations for brain tumors were registered between 2016 and 2022, all of which were included in the study. Average daily admissions for brain tumors decreased from 8.2 during the pre-pandemic period to 7.3 during the pandemic period (*p* < 0.01). Figure 1 shows weekly admissions for brain tumors within the Helios Hospital Network during pre-pandemic and pandemic years in relation to the total number of hospitalized SARS-CoV-2 infections. The corresponding trend analysis shows that, already before the pandemic, there was a consistent decrease in admissions for brain tumors. After a clear drop-off in the total number of brain tumor hospitalizations at the onset of the pandemic, this trend continued throughout all three pandemic years.

### Changes in baseline characteristics and frailty

**Fig. 2:**
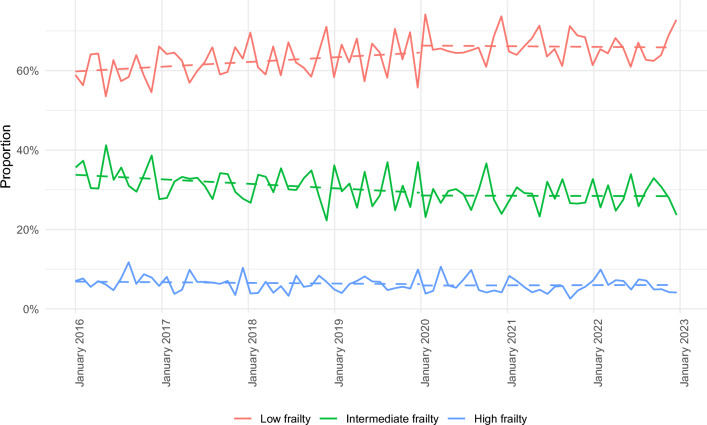
Weekly admission proportions of patients with brain tumors during pre-pandemic and pandemic periods for low (red line), intermediate (green line), and high (blue line) frailty levels. Dashed lines represent trends in weekly admission based on linear regression models

Table 1 presents baseline demographics, the distribution of HFRS, and levels of comorbidity. Cases admitted during the pandemic years were significantly older compared to pre-pandemic levels (*p* < 0.01), while there were no significant changes in patient sex. Compared to pre-pandemic levels, the median HFRS decreased substantially, from 3.1 (IQR: 0.9–7.3) in pre-pandemic years to 2.6 (IQR: 0.3–6.8) during the pandemic (*p* < 0.01). Accordingly, the mean proportion of low frailty cases increased significantly from 62.4 to 66.1% (*p* < 0.01). At the same time, the rate of high frailty decreased from 6.2% to 5.5%, as did the rate of intermediate frailty, from 31.4% to 28.4% (*p* < 0.01). A detailed depiction of frailty trends is presented in Fig. 2. Already in pre-pandemic years 2016 through 2019, the proportion of cases with low frailty levels increased consistently, yet still remained below the proportion of low frailty patients during pandemic years 2020 through 2022. At the same time, the proportion of cases with high frailty barely changed, independent of the pandemic. The mean ECI decreased from 17.0 (± 12.4) in the pre-pandemic period to 16.1 (± 12.0) during the pandemic (*p* < 0.01). When compared to low-frailty cases and to pre-pandemic levels, the observed decrease in ECI was significantly larger among high frailty cases (0.6 vs. 0.3 points; *p* = 0.04).

**Table 1:** Baseline demographics and levels of frailty and comorbidity

		Pre-pandemic period (*n* = 12,026)	Pandemic period (*n* = 7,979)	*p*
Age	Mean ± SD	59.0 ± 18.4	60.0 ± 18.4	< 0.01
Sex	Male, *n* (%)	6114 (50.8)	4007 (50.2)	
	Female, *n* (%)	5912 (49.2)	3972 (49.8)	0.40
Hospital frailty risk score	Median (IQR)	3.1 (0.9–7.3)	2.6 (0.3–6.8)	< 0.01
Low frailty	% (n)	62.4 (7,502)	66.1 (5,271)	< 0.01
Intermediate frailty	% (n)	31.4 (3,775)	28.4 (2,270)	< 0.01
High frailty	% (n)	6.2 (749)	5.5 (438)	0.03
Elixhauser comorbidity index	Mean ± SD	17.0 ± 12.4	16.1 ± 12.0	< 0.01
Low frailty	Mean ± SD	13.3 ± 10.9	13.0 ± 10.7	0.04*
Intermediate frailty	Mean ± SD	21.9 ± 12.1	21.5 ± 11.8	0.07*
High frailty	Mean ± SD	28.5 ± 12.8	27.9 ± 12.3	ref

*p values for interaction

Table 2 shows the rates of SARS-CoV-2 infections among brain tumor patients, which were in the low single digit percentages throughout the study.

**Table 2:** Number of SARS-Cov-2 infections during pandemic period

Year	SARS-CoV-2 infection, *n* (%)
2020	13 (0.49)
2021	47 (1.67)
2022	115 (4.59)

### Changes in admissions and length of stay in relation to frailty

The extent of the decrease in daily admissions was significantly larger among low frailty patients, from 5.1 to 4.8, compared to high frailty patients, from 0.5 to 0.4 (*p* < 0.01) (Table 3).

**Table 3:** Daily admissions by frailty risk group

Cohort	Pre-pandemic period	Pandemic period	*p*
Total (n)	12,026	7979	
Daily admissions	8.2	7.3	
IRR (95% CI)	0.89 (0.85–0.94)		< 0.01
Low risk (n)	7502	5271	
Daily admissions	5.1	4.8	
IRR (95% CI)	1.20 (1.05–1.38)		< 0.01*
Intermediate risk (n)	3775	2270	
Daily admissions	2.6	2.1	
IRR (95% CI)	1.03 (0.89–1.19)		0.70*
High risk (n)	749	438	
Daily admissions	0.5	0.4	
IRR (95% CI)	–		ref

Within the total group, we compared daily admissions between pre-pandemic and pandemic period. In a separate interaction analysis, we compared frailty risk groups to each other with high risk as reference. *p values for interaction between frailty groups

*IRR* incidence rate ratio

The average length of stay was significantly shorter in the pandemic (9.5 days) compared with the pre-pandemic period (10.2 days, *p* < 0.01, Table 4). This decrease in length of stay did not differ between frailty groups.

**Table 4:** Length of stay during pre-pandemic and pandemic periods per frailty risk groups

Cohort	Pre-pandemic period	Pandemic period	*p*
Total			
Mean (SD)	10.2 ± 11.8	9.5 ± 10.7	
Median (IQR)	7.0 (3, 13)	6.0 (3, 12)	
Coefficient (95% CI)	− 0.05 (− 0.07 to − 0.03)		< 0.01
Low risk			
Mean (SD)	7.2 ± 6.8	6.7 ± 6.5	
Median (IQR)	5.0 [3, 9]	5.0 [2, 8]	
Coefficient (95% CI)	− 0.02 (− 0.11 − 0.07)		0.65*
Intermediate risk			
Mean (SD)	13.2 ± 13.2	13.2 ± 12.5	
Median (IQR)	10.0 [5, 17]	10.0 [5, 17]	
Coefficient (95% CI)	0.07 (− 0.03 − 0.16)		0.18*
High risk			
Mean (SD)	25.2 ± 23.0	24.3 ± 19.1	
Median (IQR)	20.0 [12, 31]	19.0 [10, 33]	
Coefficient (95% CI)	–		ref

Within the total group, we compared the length of stay between pre-pandemic and pandemic period. In a separate interaction analysis, we compared frailty risk groups to each other with high risk as reference. *p values for interaction between frailty groups

### Changes in rates of treatment and in-hospital mortality rates in relation to frailty

Corresponding to the decrease in brain tumor admissions during the pandemic, there was a decline in the total number of conducted craniotomies (*n* = 2917) and brain tumor resections (*n* = 2391), compared to pre-pandemic levels (*n* = 3600 and *n* = 2964, respectively). However, the proportion of patients who underwent craniotomy or brain tumor resection increased significantly during the pandemic, from 29.9 to 36.6% and from 24.6 to 30.0%, respectively, with corresponding odds ratios (OR) of 1.42 (95% CI 1.33–1.52) and 1.36 (95% CI 1.27–1.45), each with *p* < 0.01 (Table 5). This effect was not associated with frailty. The rates of transfer to intensive care increased significantly during the pandemic, from 35.0 to 38.1% (OR 1.17 [95% CI 1.10–1.24]; *p* < 0.001), also without association with frailty. The total rate of mechanical ventilation did not change. However, among low frailty brain tumor patients, the rates of mechanical ventilation decreased during the pandemic, from 1.2 to 0.9%. This change was significantly different (*p* = 0.003) from that observed among high frailty patients, which displayed an increase in rates of ventilation from 18.6 to 21.7%. Rates of in-hospital mortality, which were 6.1% during the pandemic and 6.7% before, remained stable, and the differences between pandemic and pre-pandemic periods were not associated with frailty.

**Table 5:** Rates of surgery, in-hospital processes, and mortality per frailty groups

Treatment	Control period	Study period	Odds ratio (95% CI)	*p*
Craniotomy, % (*n*)	29.9 (3600)	36.6 (2917)	1.42 (1.33–1.52)	< 0.001
Low risk, % (*n*)	29.8 (2235)	36.5 (1926)	0.97 (0.72–1.31)	0.833*
Intermediate risk, % (*n*)	28.9 (1090)	35.6 (808)	1.09 (0.80–1.50)	0.580*
High risk, % (*n*)	36.7 (275)	41.8 (183)		ref
Brain tumor resection, % (*n*)	24.6 (2964)	30.0 (2391)	1.36 (1.27–1.45)	< 0.001
Low risk, % (*n*)	24.1 (1810)	29.1 (1532)	1.00 (0.75–1.35)	0.975*
Intermediate risk, % (*n*)	24.4(920)	31.1 (705)	1.21 (0.88–1.65)	0.237*
High risk, % (*n*)	31.2(234)	35.2 (154)		ref
Mechanical ventilation, % (*n*)	3.4(407)	3.3 (261)	0.97 (0.83–1.14)	0.741
Low risk, % (*n*)	1.2 (93)	0.9 (46)	0.49 (0.30–0.78)	0.003*
Intermediate risk, % (*n*)	4.6 (175)	5.3 (120)	0.87 (0.59–1.29)	0.492*
High risk, % (*n*)	18.6 (139)	21.7 (95)		ref
Intensive care, % (*n*)	35.0 (4206)	38.1 (3043)	1.17 (1.10–1.24)	< 0.001
Low risk, % (*n*)	31.7 (2376)	35.3 (1863)	1.20 (0.92–1.57)	0.186*
Intermediate risk, % (*n*)	37.9 (1432)	41.7 (946)	1.24 (0.94–1.64)	0.134*
High risk, % (*n*)	53.1 (398)	53.4 (234)		ref
In-hospital mortality, % (*n*)	6.7 (730)	6.1 (431)	0.89 (0.78–1.01)	0.070
Low risk, % (*n*)	2.0 (141)	2.3 (110)	0.98 (0.66–1.47)	0.939*
Intermediate risk, % (*n*)	13.2 (437)	12.0 (229)	0.74 (0.52–1.05)	0.094*
High risk, % (*n*)	23.2 (152)	26.1 (92)		ref

In the first line of each category, we compared pre-pandemic and pandemic period. In separate analyses, we compared frailty risk groups to each other with high risk as reference. *p values for interaction between frailty groups. Percentages presented for treatment rates represent the proportion of each type of treatment within each frailty group separately, therefore not adding up to 100%

## Discussion

In this study among 20,005 hospital admissions for brain tumors in Germany, the pandemic years 2020 through 2022 were associated with a substantial decrease in the total number of hospitalizations, compared to pre-pandemic years 2016 through 2019. During the pandemic, there was a marked decrease in overall frailty among brain tumor patients, as well as an overall decline in rates of comorbidities, which was larger among high vs. low frailty patients. Even though frailty and rates of comorbidity decreased, brain tumor patients admitted during the pandemic were significantly older by a mean of 1 year. At the same time, there was a significant increase in the rates of surgery across all levels of frailty.

Changes in the management and treatment of different types of cancer during the COVID-19 pandemic have previously been reported, mainly emphasizing how case volume and personnel were negatively impacted [20–23]. Previous studies reported that, during the pandemic, imaging procedures in brain tumor care were delayed or even canceled, and symptom-based diagnosis of cancer became more important [20, 21]. In the early phases of the pandemic, Price et al. reported significant alterations in the management of malignant brain tumors in 11% of cases [24]. Some authors have even argued in favor of initiating radiation and chemotherapy in older glioma patients without a histological diagnosis, in hope that omitting surgical intervention might prevent in-hospital infection with SARS-CoV-2 [25].

Prior to our study, associations between the COVID-19 pandemic and frailty among brain tumor patients had not been examined, although frailty is known to be associated with outcomes. Tracking longitudinal pandemic-associated changes in frailty in brain tumor patients is of interest for clinicians given that the general population has been aging at rapid rates over recent decades [26] and the current pandemic has increased frailty levels among community-dwelling older persons [7, 8].

The fact that our study identified a decrease in frailty and in the burden of comorbidity, compared to pre-pandemic levels, is in line with evidence on patients admitted for acute ischemic stroke in Germany, which also observed a decline in the prevalence of comorbidities during the pandemic [27].

Previous evidence suggests that higher levels of frailty among older brain tumor patients with underlying comorbidities are associated with increased length of stay and higher rates of mortality [12]. Given that, in our study, the pandemic was associated with improved frailty and lower rates of comorbidities among brain tumor patients, it is unsurprising that the length of stay decreased and mortality rates did not rise, compared to pre-pandemic levels. Furthermore, it is reasonable to assume that, during the COVID-19 pandemic, health care professionals may have attempted to keep the length of stay as low as possible in an attempt to provide hospital bed vacancies for potentially arriving COVID-19 patients. Such organizational changes during the pandemic may have impacted other in-hospital processes, as well. Furthermore, the fact that the total number of admissions for brain tumors had been continuously decreasing already prior to the onset of the pandemic is most likely due to ongoing restructuring processes within the German health system toward the provision of more out-patient care. Given that there were no substantial changes in guidelines for brain tumor care or in screening methods for brain tumors during our study period, such organizational developments may have played a significant role in the observed changes in hospitalizations and also in frailty levels during the pandemic.

Previous studies also suggest that, during the pandemic, early discharge was enforced with the aim to reduce the risk of COVID-19 exposure for non-COVID-19 patients [28]. This may have played a role in our study, as well. Another important factor may have been that infections with SARS-CoV-2 were rare in our patient cohort. Therefore, the well-established unfavorable association between frailty, SARS-CoV-2 infections, and mortality rates may not have materialized to relevant degrees in our study [29, 30].

When discussing our finding that, during the pandemic, rates of surgery increased, it is important to note that a less frail brain tumor patient population with a lower burden of comorbidities, as observed in our study during the pandemic, is more likely to qualify for surgery. Previous evidence suggests that rates of surgery may have risen during the pandemic due to an increased prevalence of tumor-related symptoms [24] or by patients with mild symptoms having been discharged during the pandemic prior to potential surgery [31]. Interestingly, our results suggest that neurosurgeons in Germany were not influenced by frailty levels when deciding on whether to operate, given that the observed increase in rates of surgery were uniform across all three examined frailty groups. This is different from spine surgery, in which, during the pandemic, in Germany, less frail patients were more likely to undergo surgery [32].

Our study is the first to describe changes in frailty among patients hospitalized for brain tumors during the COVID-19 pandemic. Given that our findings suggest that a relevant proportion of high frailty brain tumor patients may have avoided hospitalization during the pandemic, our study points to the importance of creating environments outside of hospitals in which high frailty brain tumor patients can be supported. The beneficial role of Advanced Clinical Practitioners (ACPs), which are experienced and registered health care professionals, during the COVID-19 outbreak in England is a good example for how, in times of crisis, more creative, personalized, and sustainable solutions may be implemented in the care for older people living with frailty [33]. Evidence shows that, during the pandemic, the work of ACPs in the domains of clinical practice, education, leadership, and research was a crucial contributor to healthcare in England [34].

Our study has several limitations. First, even though most clinical studies measuring frailty in general or even according to the HFRS were conducted in patient cohorts comprising all ages, like in our study, it is worth noting that the HFRS has only been validated in patients ≥ 75 years of age. Therefore, in subjects younger than 75 years in our study, the measured frailty levels may be more representative of the burden of comorbidity rather than frailty itself. Second, since we analyzed administrative data of 78 hospitals, inhomogeneous encoding information among hospitals could lead to misclassification of ICD-10 and OPS codes. Yet, all codes underwent rigorous in-hospital auditing before entry into the database. Third, we did not exclude conditions frequently associated with brain tumors, such as neurological deficits, which may introduce some selection bias to our study, given that the HFRS also includes some neurological deficits as part of its coding structure. Fourth, given that the first wave of the pandemic did not arrive before March 2020, having added hospitalizations from January and February 2020 to the “pandemic years” cohort may confound our findings. Fifth, our analysis is not granular enough to differentiate changes in frailty during separate phases of the pandemic, such as the beginning versus later waves. Also, due to the administrative nature of the data, double inclusions of patients admitted more than once during the study period cannot be ruled out. Furthermore, our study did not include information on initial neurological status, medication, tumor size, histopathological findings, or metastasis to other parts of the body. Also, some of the changes in brain tumor care observed during the pandemic, such as decreased length of stay or increased rates of transfer to intensive care, may have partially been influenced by alterations in in-hospital processes unrelated to the pandemic situation. Finally, given that all data stem from Germany, generalizability to other countries may be limited.

## Conclusion

Even though our findings are limited in that the HFRS is validated only for patients  ≥  75 years of age, our study among patients of all ages hospitalized for brain tumors in Germany suggests a marked decrease in levels of frailty and in the burden of comorbidities during the COVID-19 pandemic. This suggests a selection for healthier individuals and may be one of the main contributing factors to increasing rates of surgery, compared to pre-pandemic levels. Given an ever-aging population, awareness of pandemic-associated trends in frailty among brain tumor patients and related longitudinal trends in brain tumor care are of paramount importance to clinicians and healthcare providers to prepare for future challenges.

## Data Availability

All data were stored and analyzed in pseudonymized form.

## Code availability

The datasets generated during and/or analyzed during the current study are available from the corresponding author on reasonable request.
